# A Novel Role for the BMP Antagonist Noggin in Sensitizing Cells to Non-canonical Wnt-5a/Ror2/Disheveled Pathway Activation

**DOI:** 10.3389/fcell.2017.00047

**Published:** 2017-05-04

**Authors:** Ondrej Bernatik, Tomasz Radaszkiewicz, Martin Behal, Zankruti Dave, Florian Witte, Annika Mahl, Nicole H. Cernohorsky, Pavel Krejci, Sigmar Stricker, Vitezslav Bryja

**Affiliations:** ^1^Faculty of Sciences, Institute of Experimental Biology, Masaryk UniversityBrno, Czechia; ^2^Institute for Chemistry and Biochemistry, Freie Universität BerlinBerlin, Germany; ^3^Department of Biology, Faculty of Medicine, Masaryk UniversityBrno, Czechia; ^4^Department of Cytokinetics, Institute of Biophysics AS CR, v.v.i.Brno, Czechia

**Keywords:** noggin, Wnt5a, non-canonical Wnt pathways, BMP signaling, brachydactyly, Ror2

## Abstract

Mammalian limb development is driven by the integrative input from several signaling pathways; a failure to receive or a misinterpretation of these signals results in skeletal defects. The brachydactylies, a group of overlapping inherited human hand malformation syndromes, are mainly caused by mutations in BMP signaling pathway components. Two closely related forms, Brachydactyly type B2 (BDB2) and BDB1 are caused by mutations in the BMP antagonist Noggin (NOG) and the atypical receptor tyrosine kinase ROR2 that acts as a receptor in the non-canonical Wnt pathway. Genetic analysis of Nog and Ror2 functional interaction via crossing Noggin and Ror2 mutant mice revealed a widening of skeletal elements in compound but not in any of the single mutants, thus indicating genetic interaction. Since ROR2 is a non-canonical Wnt co-receptor specific for Wnt-5a we speculated that this phenotype might be a result of deregulated Wnt-5a signaling activation, which is known to be essential for limb skeletal elements growth and patterning. We show that Noggin potentiates activation of the Wnt-5a-Ror2-Disheveled (Dvl) pathway in mouse embryonic fibroblast (MEF) cells in a Ror2-dependent fashion. Rat chondrosarcoma chondrocytes (RCS), however, are not able to respond to Noggin in this fashion unless growth arrest is induced by FGF2. In summary, our data demonstrate genetic interaction between Noggin and Ror2 and show that Noggin can sensitize cells to Wnt-5a/Ror2-mediated non-canonical Wnt signaling, a feature that in cartilage may depend on the presence of active FGF signaling. These findings indicate an unappreciated function of Noggin that will help to understand BMP and Wnt/PCP signaling pathway interactions.

## Introduction

Limb bud development and the concomitant formation of limb skeletal structures are regulated by the intricate interplay and integration of various signaling pathways, with major roles played by the Shh, BMP, FGF, and Wnt/β-catenin pathways (reviewed for example in, Robert, 2007; Zuniga, 2015). The BMP signaling pathway is of pivotal importance especially for skeletal development. The analysis of inheritable human hand malformation syndromes has been instrumental in understanding the contribution of BMP signaling and other pathways for skeletal development. One example are the brachydactylies, a group of inheritable syndromes that are characterized by shortening or absence of phalanges. Most brachydactyly subtypes are caused by mutations in BMP signaling components or factors that, at different levels, intersect with BMP signaling. Therefore brachydactylies have been interpreted in terms of a molecular disease family (Stricker and Mundlos, 2011). This hypothesis predicts that overlapping phenotypes are likely caused by mutations affecting components that show a close functional interaction within a common signaling network.

Intriguingly, two closely related brachydactyly subtypes, BDB1 and BDB2, are caused by mutations in ROR2 or NOGGIN, respectively (Oldridge et al., 2000; Lehmann et al., 2007). While NOG is well known as a secreted BMP antagonist, ROR2 is an atypical receptor tyrosine kinase that is involved in the inhibition of Wnt/β-catenin signaling (Mikels and Nusse, 2006). In developing digits, Ror2-mediated Wnt/β-catenin inhibition allows BMP-mediated digit outgrowth (Witte et al., 2010). In addition, Ror2 is a Wnt (co)receptor, mainly for Wnt-5a, acting in non-canonical Wnt signaling (Oishi et al., 2003; Schambony and Wedlich, 2007). Recently, activation of the non-canonical Wnt/planar cell polarity (PCP) pathway by Wnt-5a and ROR2 was shown to be critically involved in the regulation of limb skeleton development (Gao et al., 2011; Wang et al., 2011; Ho et al., 2012; Kuss et al., 2014). Moreover, a separate set of mutations in ROR2 causes autosomal recessive Robinow syndrome (RS), which is characterized by diverse malformations including the axial and limb skeleton (Afzal et al., 2000; van Bokhoven et al., 2000). A dominant form of RS is caused by mutations in Wnt/PCP components DVL1, DVL3, and WNT-5A, it is therefore believed that the developmental defects seen in Robinow syndrome are caused by a deregulation of Wnt-5a/Ror2/PCP signaling (Stricker et al., 2017).

The skeletal elements of the limbs are formed by endochondral ossification. In this process a cartilage template is formed that mediates growth of the skeletal element and becomes later replaced by bone. This process is dependent on the formation of stacked columns of proliferating chondrocytes oriented perpendicular to the longitudinal axis of the growing skeletal element (Romereim and Dudley, 2011). Deregulation of PCP signaling in proliferating chondrocytes leads to perturbation of column formation, and to arbitrary chondrocyte orientation that ultimately leads to skeletal malformations typically resulting in a shortening and widening of the skeletal elements (Ahrens et al., 2009; Li and Dudley, 2009; Kuss et al., 2014; Romereim et al., 2014).

Based on the close phenotypic overlap of human brachydactyly-causing mutations in ROR2 and NOG, we hypothesized that NOG may directly interact with the Wnt-5a/Ror2 pathway. We show here a subtle genetic interaction of Noggin with Ror2 during mouse limb development. Mechanistically, we provide evidence that Noggin can sensitize cells to Wnt/PCP pathway activation mediated by ROR2, providing first evidence for a yet uncharacterized level of cross-talk between BMP and Wnt/PCP signaling.

## Materials and methods

### Mouse lines and phenotypical analysis

Ror2^+/−^ (Takeuchi et al., 2000) and Nog^+/−^ (McMahon et al., 1998) were maintained as heterozygous lines and intercrossed to yield compound mutants. Timed matings were set up and embryos were collected at E18.5. Skeletal preparations were performed as described previously (Mundlos, 2000). All animal procedures were carried out in accordance with European Union and German law. Animals were maintained in the SPF mouse facility of the Max Planck Institute for Molecular Genetics, Berlin under license from the Landesamt für Gesundheit und Soziales (LAGeSo) under license numbers ZH120 and G0346/13.

### Cell culture and treatments

Ror1^−/−^ Ror2^−/−^ mouse embryonic fibroblasts (MEF) were derived from Ror1 ^flox/flox^ Ror2 ^flox/flox^ MEF cells as described previously (Ho et al., 2012). MEF and RCS cells were propagated in DMEM, 10% FCS, 2 mM L-glutamine, 50 units/ml penicillin, and 50 units/ml streptomycin. RCS cells were seeded in 24-well plates, grown for 24 h and treated as indicated. Following reagents: Wnt-5a (R&D systems, 645-WN-010), Noggin (R&D Systems, 1967-NG-025), FGF2 (5 ng/ml, R&D Systems) and Wnt-C59 5 μM (Tocris Bioscience, 5148) were used for treatment. Wnt-5a conditioned media was produced from L Wnt-5a cells (ATCC CRL-2814) according to ATCC instructions. RCS cells intended for WB analysis were treated by FGF2 for 48 h, then were treated by the porcupine inhibitor Wnt-C59 (to reduce background autocrine Wnt activity), Noggin and Wnt-5a in indicated doses for additional 24 h. Total time of FGF2 treatment was 72 h.

### Western blotting

Lysates for western blotting were prepared as follows: Growth medium was removed and cells were directly lysed in 100 mM Tris/HCl (pH 6.8), 20% glycerol, 1% SDS, 0.01% bromophenol blue and 1% 2-mercaptoethanol. Western blotting was performed according to manufacturer's instructions with minor adjustments [SDS-PAGE run on 150 V, transfer onto PVDF membrane 1 h on 100 V, both steps on ice (BIO-RAD)]. Antibodies were from Santa Cruz Biotechnologies: anti-Dvl2 (dephospho-Dvl2)–sc8026, anti-beta-Actin–sc1615-R, anti-Dvl3 sc8027 and from Cell Signaling Technologies: anti-Dvl2–CS3224. Anti-Ror2 antibody was a gift from Henry Ho (UC Davis) (Ho et al., 2012). Phosphorylation status of Dvl2 and Dvl3 was quantified by densitometric analysis of Western Blot in three independent replicates using Fiji distribution of ImageJ software as described (Bernatik et al., 2014). For pDvl/Dvl rations the peak area for the upper band representing P-Dvl was divided by the peak area of the lower band (Dvl). Data was analyzed by paired *t*-test (GraphPad Prism).

### Dual Luciferase assay

RCS cells were transfected using pRLtkLuc and Super8X TopFlash plasmid. 9μg Super8X TopFlash and 3 μg pRLtkLuc plasmid were mixed with 38.4 μl of Fugene6 (E2691, Promega) in 1200 μl of DMEM. Cells were treated by 0.3% collagenase type II (GIBCO, cat.no.17101015) before transfection, 50 μl of transfection mixture and 500 μl of collagenase treated RCS cells in DMEM were used per 1 well of 24 well plate. Transfection was carried out overnight, cells were treated according to the experimental scheme for 20 h, and samples were processed by Dual-Luciferase® Reporter Assay System according to the manufacturer instructions (Promega, E1960).

## Results

### Noggin genetically interacts with Ror2

**Figure 1:**
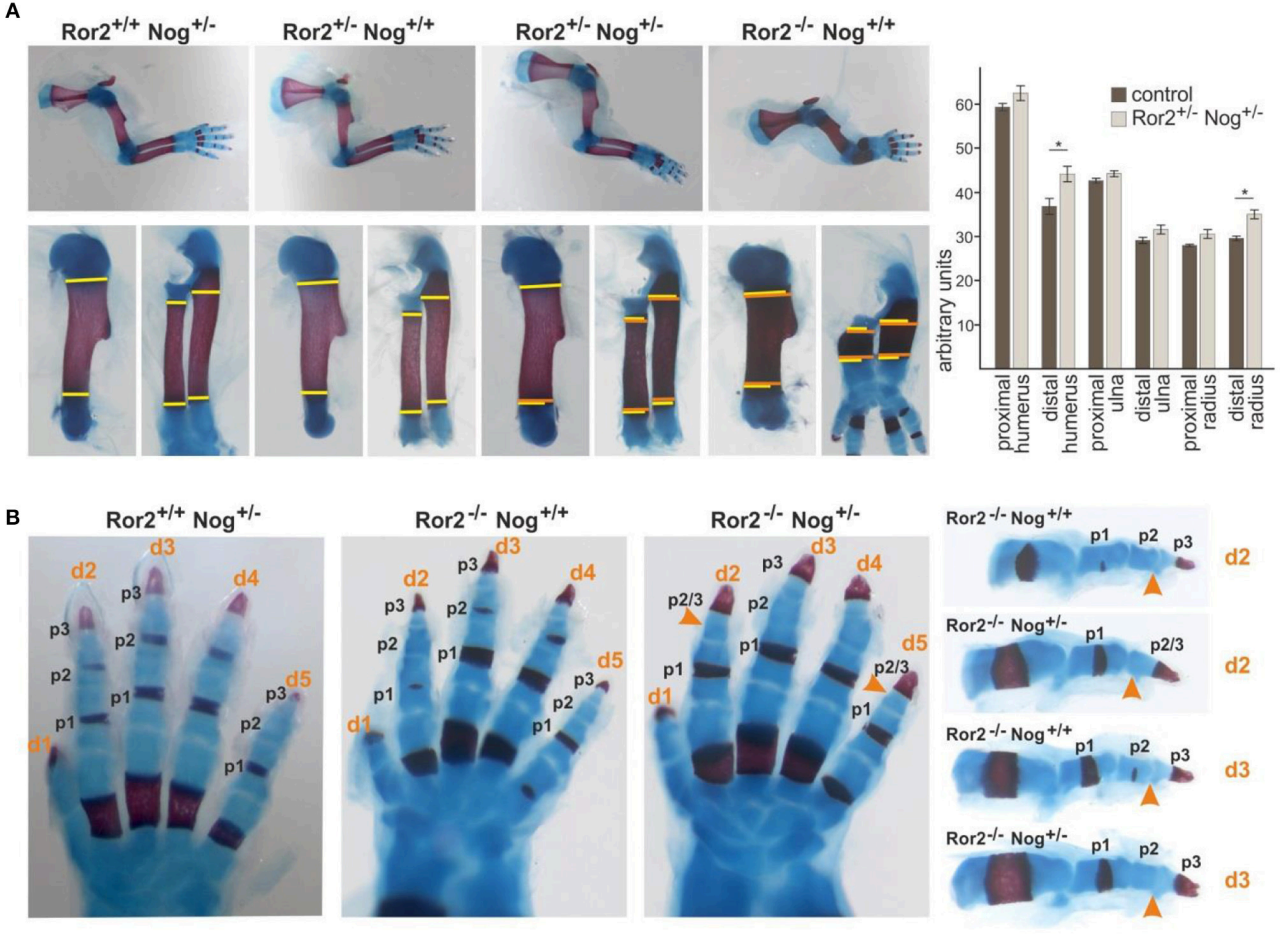
**Noggin genetically interacts with Ror2**. Skeletal preparations of E18.5 embryos of the indicated allelic combinations are shown. Cartilage stains blue, bone stains red. **(A)** Top panel: Limbs of compound Ror2 and Noggin heterozygous mutants have a normal appearance. Ror2^−/−^ skeletal elements are visibly shortened and enlarged. Bottom panel: magnifications of humerus and radius/ulna. The width of the wild type or single heterozygous skeletal elements is indicated by a yellow line on either side of the ossification center. Width of the double heterozygous or Ror2^−/−^ skeletal elements is indicated by orange line for comparison. A quantification of skeletal element width is shown right; significant effects were observed for the distal humerus and distal radius (*p* < 0,05; student's *t*-test). **(B)** Digit development in compound mutants. Ror2^−/−^ digits are shortened, but individual phalanges (p1, p2, and p3) are present, separated by synovial joints. In Ror2^−/−^;Nog^+/−^ animals, the medial phalange (p2) shows additional shortening, which in digits 2 and 5 leads to distal symphalangism of p2 and p3.

To get a first indication whether Ror2 and Noggin might functionally interact we generated compound mutants for Ror2 and Noggin. Ror2^+/−^ mice (Takeuchi et al., 2000) were crossed to Noggin^+/−^ mice (Brunet et al., 1998; McMahon et al., 1998). Heterozygous inactivation of either Ror2 or Noggin does not result in any skeletal alteration (Figure 1A). In Ror2^+/−^;Nog^+/−^compound heterozygotes the overall appearance of the limb skeleton was normal; however the skeletal elements of the stylopod (the humerus) and the zeugopod (radius and ulna) showed a consistent small lateral expansion (Figure 1A, width of skeletal elements in wild type and single mutants indicated in yellow, width in compound mutant indicated in orange). All skeletal elements showed a tendency toward widening at both metaphyseal sides, however statistical significance was only reached for the distal humerus and radius, respectively. This feature was not seen in single heterozygotes, indicating genetic interaction between *Nog* and *Ror2*.

Ror2^−/−^ mice are a model for RRS, recapitulating several of its features including mesomelic limb shortening as well as mild brachydactyly (Schwabe et al., 2004). Ror2^−/−^ mice have shortened digits, however all phalanges (two in the thumb/digit 1, three in digits 2–5) as well as the interphalangeal joints separating the phalanges are present (Takeuchi et al., 2000; Schwabe et al., 2004; Schwarzer et al., 2009) (Figure 1B). Noggin heterozygous mice have phenotypically normal digits. When one allele of Noggin was removed on the Ror2^−/−^ background, shortening of phalanges was further increased. In digit 3 the appearance of 3 individual phalanges, which were smaller than those in the Ror2^−/−^, was preserved. In digits 2 and 5 loss of one Noggin allele on the Ror2^−/−^ background led to loss of an individual phalanx 2, concomitant with a longer phalanx 3, indicating failure of distal joint formation. Distal joint fusion is also a feature seen sometimes in BDB1 (ROR2 mutation) and frequently in BDB2 (NOG mutation). In addition, joint fusions are the hallmark of proximal symphalangism 1A (SYM1A) and multiple synostosis syndrome (SYSN1), two conditions caused by a different set of NOG mutations (Stricker and Mundlos, 2011). Altogether the compound mutants support the notion of a genetic and functional interaction of Ror2 and Nog in skeletal development.

### Noggin potentiates Wnt/PCP signaling in a Ror2-dependent manner

**Figure 2:**
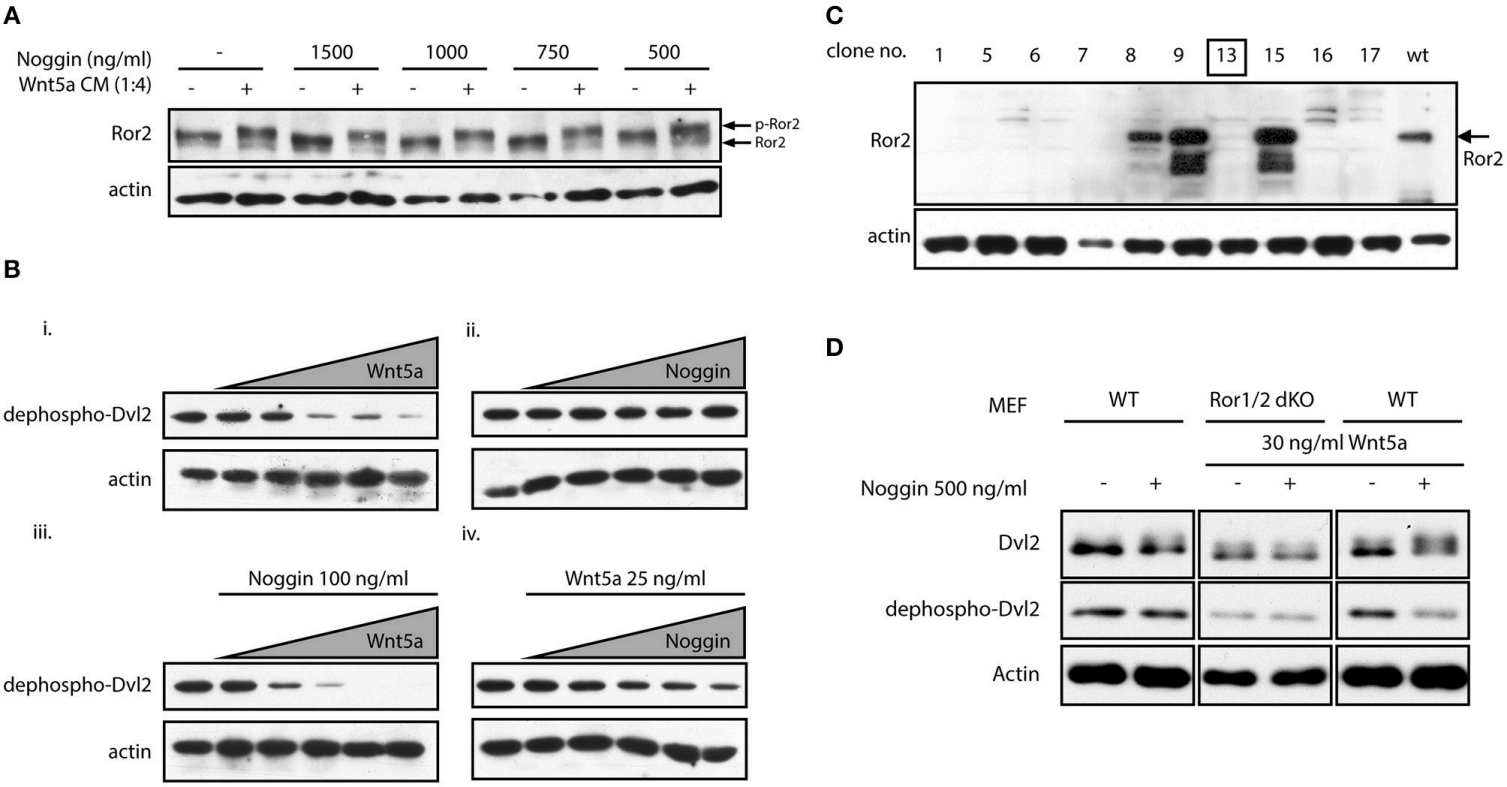
**Noggin potentiates Wnt/PCP signaling. (A)** MEF cells were treated by Wnt-5a conditioned medium (CM) and stimulated by increasing doses of Noggin protein. Activation of Ror2 was analyzed as a phosphorylation-dependent shift by Western blotting. Noggin alone, in contrast to Wnt-5a CM, is not able to trigger activation of Ror2. **(B)** MEF cells were treated with increasing doses of Wnt-5a (0, 25, 50, 100, 150, and 200 ng/ml) and Noggin (0, 25, 50, 100, 150, and 200 ng/ml) for 2 h. The activation of Wnt signaling was assessed by Western blotting as a decrease in the signal of dephospho-Dvl2. Wnt-5a could cause phosphorylation of Dvl2 visible as a disappearance of dephospho-Dvl2 signal **(i)**, whereas Noggin is inactive in the same assay **(ii)**. Interestingly, pre-treatment of MEF cells by Noggin (100 ng/ml) enhanced the effect of Wnt-5a **(iii)**. On the other hand, Noggin, in a dose-dependent manner, potentiated the response to suboptimal doses of Wnt-5a (25 ng/ml), which are otherwise ineffective—see lane 2 in panel “i” **(iv)**. Actin is used as a loading control. **(C)** *Ror1*^flox/flox^; *Ror2*^flox/flox^ MEF cells were treated by tamoxifen and *Ror1*^−/−^*; Ror2* ^−/−^ isogenic MEF line was isolated by serial dilutions method. The presence of Ror2 was tested by Western blotting and the clone no. 13 used for further studies is indicated. **(D)** MEF wt and MEF *Ror1*^−/−^*; Ror2*^−/−^ (Ror1/2 dKO) cells were treated by combinations of Noggin and Wnt-5a as indicated. Noggin itself cannot stimulate activation of Dvl2—visible as a phosphorylation-dependent shift of Dvl2 (upper blots) or decrease in dephospho-Dvl2 (middle blots) signal. Noggin, however, increases activity of suboptimal dose of Wnt-5a (30 ng/ml), an effect that is lost in *Ror1*^−/−^ *Ror2*^−/−^ MEF cells.

In digit formation, Ror2 acts in part via inhibition of β-catenin signaling leading to derepression of BMP/SMAD signaling in a structure called phalanx-forming region (Witte et al., 2010). Evidence however has accumulated that in addition or in parallel to this function Ror2 and its paralog Ror1 are both required for Wnt-5a/PCP signaling activation during digit development (Gao et al., 2011; Ho et al., 2012). Our genetic interaction experiments cannot distinguish the origin of the interaction seen, i.e., whether it originated from Nog function in the BMP pathway, or a yet uncharacterized role in the Wnt-5a/PCP pathway. Noggin thus might not only influence activity of BMP, but also of Wnt-5a-Ror2 pathway. To test if Noggin is able to activate Ror2 we treated mouse embryonal fibroblasts (MEF) with increasing doses of Noggin. The activation of endogenous Ror2 can be monitored as a phosphorylation-dependent mobility shift on Western blotting (Oishi et al., 2003). As we show in Figure 2A, even in the highest concentrations used (1,500 ng/ml) Noggin did not induce phosphorylation of Ror2 and was unable to promote phosphorylation of Ror2 induced by its cognate ligand Wnt-5a. This suggests that at the receptor level Noggin is unable to act either directly as a ligand for Ror2, or indirectly.

In the next step we tested if Noggin can promote any of the Ror2-downstream events. A robust readout of non-canonical Wnt pathways activation is the Wnt-5a-induced phosphorylation of Disheveled (Dvl) 2, an event dependent on the Ror1 and Ror2 receptors (Ho et al., 2012). We took advantage of an anti-Dvl2 antibody that recognizes only the inactive, dephosphorylated form of Dvl2 in MEF cells (Gonzalez-Sancho et al., 2013). Disappearance of non-phosphorylated Dvl2 currently represents one of the most sensitive tools for visualization of Dvl2 phosphorylation and hence Wnt/PCP pathway activation. When we treated MEF cells with increasing doses of Wnt-5a, the non-phospho Dvl2 signal disappeared (Figure 2Bi), indicative of activated Wnt-5a-Ror-Dvl signaling. No such phenotype was observed when cells were treated by Noggin, confirming our previous observation that Noggin itself is not able to activate signaling via Ror2 (Figure 2Bii). However, when cells were treated with 100 ng/ml of Noggin, we could clearly observe stronger effects of Wnt-5a on Dvl2 activation (compare Figure 2Bi vs. Figure 2Biii). This indicates that Noggin can sensitize MEF cells to Wnt-5a/Ror2 signaling. To confirm this observation, we treated cells with 25 ng/ml of Wnt-5a, which is a suboptimal dose unable to trigger Dvl2 activation (Figure 2Bi). When cells pre-treated by 25 ng/ml of Wnt-5a were supplemented with increasing doses of Noggin, activation of Dvl2 was observed in a dose dependent manner (Figure 2Biv), indicating that presence of Noggin can reveal biological activity of previously sub-threshold Wnt-5a concentrations.

All these data suggest that Noggin, despite its inability to activate Ror2 on its own, can efficiently potentiate the Wnt-5a-Ror2 signaling axis and sensitize cells to low amounts of Wnt-5a. Ror2 can have redundant function with closely related Ror1 (Ho et al., 2012) that can also bind Wnt-5a. To confirm that the effects of Noggin are indeed dependent on Ror1/Ror2, Ror1^−/−^ Ror2^−/−^ double knockout MEF cells were isolated from conditional Ror1/Ror2 knockout mice (as described in Ho et al., 2012). Individual clones were tested by Western blotting (Figure 2C) and one of the Ror1/Ror2 double negative clones (#13) was further used for functional analysis. When Ror1/Ror2-deficient MEF cells were treated with 30 ng/ml of Wnt-5a and 500 ng/ml of Noggin simultaneously, no shift of Dvl2 mobility (upper panels) or effects on non-phospho Dvl2 (middle panel) was observed, in contrast to wt MEF where Dvl2 was activated by the combination of Wnt-5a (30 ng/ml) and Noggin (500 ng/ml) (Figure 2D). This data show that Noggin is able to potentiate the activation of the Wnt-5a-Ror2 signaling circuit and demonstrate that the observed Noggin/Wnt-5a synergism toward Dvl2 is dependent on Ror1/Ror2.

### FGF2-induced chondrocyte growth arrest enables Noggin-mediated Wnt/PCP potentiation in RCS cells

The genetic interaction between Ror2 and Noggin observed in mice as well as the skeletal involvement in human syndromes characterized by NOG and ROR2 mutations pointed toward the importance of a functional Noggin-Ror2 interaction for skeletal development. To test the Noggin-Ror2 synergy in a model system that is more relevant to skeletal development we decided to use the rat chondrosarcoma (RCS) cell line. RCS chondrocytes maintain a fully differentiated proliferating chondrocyte phenotype in culture, manifested by abundant production of cartilaginous extracellular matrix rich in sulfated proteoglycans and collagen type 2, but not collagen type 10 characteristic for hypertrophic chondrocytes (Mukhopadhyay et al., 1995). Moreover, RCS chondrocytes faithfully recapitulate FGF-receptor 3 (FGFR3) signaling in the growth plate cartilage. Many essential features of FGFR3 signaling in the growth plate cartilage, such as the FGF-mediated chondrocyte growth-arrest have been unraveled using the RCS chondrocyte model system (Aikawa et al., 2001; Dailey et al., 2003; Krejci et al., 2005).

**Figure 3:**
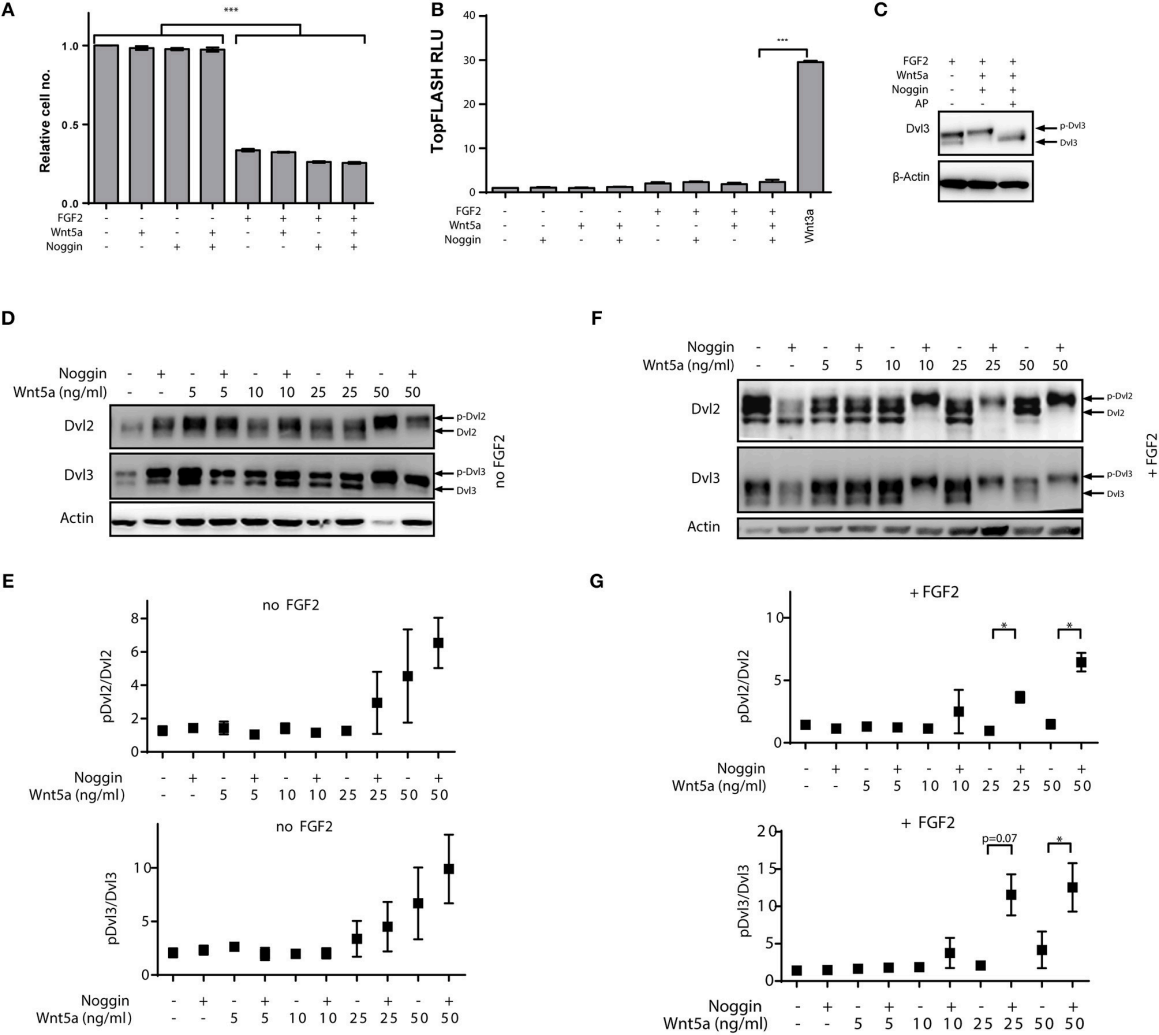
**Positive effects of Noggin on Wnt/PCP activation in chondrocytes is induced by co-stimulation with FGF2**. RCS cells were treated by FGF2 to induce growth arrest, and then treated by the porcupine inhibitor Wnt-C59 (5 μM) to reduce the autocrine Wnt activity and by Noggin and Wnt-5a as indicated. Timepoints are specified in Materials and Methods section. **(A)** Wnt-5a, Noggin and their combination does not alleviate growth arrest of RCS cells induced by FGF2 (72 h), graph shows average and SD from two independent experiments, ^***^p < 0.001 [One-way ANOVA (ANalysis Of VAriance) with *post-hoc* Tukey test]. (**B)** Treatment of RCS cells by FGF2, Noggin and Wnt-5a does not activate canonical Wnt pathway analyzed by TopFlash reporter system. Treatment with Wnt3a was used as a positive control. RLU—relative light units, graph shows average and SD from two independent experiments, ^***^p < 0.001 [One-way ANOVA (ANalysis Of VAriance) with *post-hoc* Tukey test]. **(C)** Alkaline phosphatase (AP) treatment can remove the electrophoretic mobility shift of Dvl3 induced by FGF2/Noggin/Wnt-5a treatment, which suggests that the mobility changes (used in **D–G**) are caused by phosphorylation. **(D)** Wnt-5a can activate downstream signaling—visible as phosphorylation-dependent shift (p-Dvl) of Dvl2 and Dvl3—at 50 ng/ml and this effect is not positively modulated by the addition of Noggin (125 ng/ml). **(E)** Quantification of p-Dvl/Dvl ratios for Dvl2 and Dvl3 from three independent experiments. **(F)** Similar experiment as in **(D)** but RCS cells were pre-treated also by FGF2 (5 ng/ml) for total 72 h to induce FGFR3-mediated growth arrest. Under these conditions 24 h treatment by C59, Noggin (125 ng/ml) and Wnt-5a can dramatically induce the Wnt-5a-induced activation of Dvl2 and Dvl3. **(G)** Quantification of three independent experiments. ^*^p < 0.01 (paired *t*-test).

To define this experimental system, we first investigated whether the FGF-induced growth arrest in RCS cells is influenced by addition of Noggin and Wnt-5a. Noggin, Wnt-5a and/or their combination did not induce a growth arrest by themselves, and also did not modulate the FGF-induced growth arrest of RCS cells (Figure 3A). We also wanted to exclude that any possible observations in RCS cells are caused by modulation of canonical Wnt pathway that was shown to oppose Wnt/PCP pathway in chondrogenesis. Since it was shown that RCS cells are responsive to canonical Wnt ligands, e.g., Wnt3a (Krejci et al., 2012), we tested whether Noggin and Wnt-5a treatment alters the canonical Wnt pathway in RCS cells using TopFlash reporter assay. These results (Figure 3B) showed that Noggin and Wnt-5a could not activate or inhibit the canonical Wnt pathway even though RCS cells responded well to canonical Wnt ligands such as Wnt-3a (Figure 3B). We conclude that combined treatment of RCS cells with Noggin/Wnt-5a does not influence FGF2 induced growth arrest or the canonical Wnt signaling pathway in RCS cells.

Finally, we analyzed whether RCS cells respond to combined Noggin/Wnt-5a treatment similarly to MEF cells. As we could not detect any signal by using the dephospho-Dvl2 antibody used in MEF cells (not shown), we have used an alternative readout—electrophoretic mobility shift of Dvl induced by Wnt-5a. Such mobility shift indeed represents a phosphorylation and can be effectively abrogated by alkaline phosphatase (AP) treatment (Figure 3C). Using this readout we next tested whether Noggin could potentiate the response to Wnt-5a in RCS cells similarly as was observed in MEF cells. When RCS cells were treated by combination of Wnt-5a and Noggin, no potentiation of Wnt-5a-Ror2 signaling was observed (Figure 3D, quantified in Figure 3E), and only the highest dose of Wnt-5a triggered phosphorylation of Dvl2 and Dvl3. However, when RCS cells were pre-treated with FGF2 for 2 days in order to induce growth arrest (Krejci et al., 2010), Noggin dramatically improved the response of RCS cells to low doses of Wnt-5a (Figure 3F, quantified in Figure 3G). Importantly, acute treatment of RCS cells with FGF2, Noggin and Wnt-5a was unable to induce such “sensitization” (data not shown). These data thus argue that the synergism between Noggin and Wnt-5a-Ror2 is not a proximal effect of FGF2-induced signaling or an inhibition of the canonical branch of Wnt signaling but is rather induced by cell changes caused by prolonged FGF2 treatment and cell cycle arrest.

## Discussion

Signaling pathways do not operate as standalone units but functionally cooperate and interact. Inspired by the phenotypic resemblance of BDB1 and BDB2, inheritable syndromes caused by mutations in ROR2 or NOGGIN, respectively, we decided to study how Noggin, an inhibitor of BMP pathway, and non-canonical Wnt signaling, driven by Ror2 receptor, can interact. We could show that Noggin increased biological activity of Wnt-5a and rendered cells sensitive to Wnt-5a concentrations otherwise not causing cellular responses. This function was dependent on the presence of Ror2, but Noggin did not elicit a signal on its own via Ror2.

Our study does not elucidate the molecular mechanism behind this interaction. One mechanism may involve BMP receptor type 1 b (Bmpr1b), which is mutated in BDA2 (Lehmann et al., 2003). *In vitro*, Ror2 and Bmpr1b were shown to interact and Ror2 is phosphorylated by Bmpr1b (Sammar et al., 2004, 2009). The functional consequence of this phosphorylation remains unclear but one can speculate that the effects of Bmpr1b on Ror2 are controlled by BMP ligands, whose active concentration is controlled by Noggin. Another possibility, which we were, however, not able to prove (data not shown) can be formation of Noggin-Wnt-5a-Ror2 ternary complex with the increased signaling capacity in comparison to Wnt-5a-Ror2 only. As another alternative, Noggin can, via regulation of BMP pathway, control signaling competence or cell surface amount of Ror2—here a possible point of crosstalk can be represented by Smurf family E3-ligases, which were reported to control both BMP pathway (negatively) as well as Wnt/PCP pathway (positively) (Narimatsu et al., 2009).

The importance of the BMP pathway and its tight regulation by antagonists for digit development is underscored by the fact that the majority of human brachydactylies are caused by mutations in different members of this signaling network (reviewed in Stricker and Mundlos, 2011). A necessity for integration of BMP and Wnt/β-catenin pathways has been reported for numerous developmental processes (Itasaki and Hoppler, 2010). For example, in digit outgrowth, BMP/SMAD signaling is fine-tuned by inhibition from the Wnt/β-catenin pathway, which itself is kept in check by Ror2 (Witte et al., 2010). Non-canonical (or alternative) Wnt pathways regulate entirely different aspects of tissue development compared to the Wnt/β-catenin pathway, but are connected with the BMP pathway as well, albeit the connection has not been studied to the same depth (Narimatsu et al., 2009; Schille et al., 2016). In developing limbs, Wnt/PCP signaling was involved in both digit shaping and outgrowth (Gao et al., 2011; Wang et al., 2011; Ho et al., 2012). Altogether this substantiates that both BMP and non-canonical Wnt pathways are required and act in concert during the establishment of the limb skeleton. Ror2 appears to be a pivotal intersection point between these two pathways.

Our work on RCS chondrocytes, a cell model for chondrocyte growth and differentiation that to some extent recapitulate the behavior of developing limb growth plate cartilage (Krejci et al., 2012) showed that Noggin could potentiate Wnt-5a-Ror2 pathway activity much more effectively when growth arrest was induced by FGF2 stimulation. It was previously shown in RCS chondrocytes that the FGF pathway can stimulate phosphorylation of LRP6, a co-receptor of the Wnt/β-catenin pathway (Krejci et al., 2012; Buchtova et al., 2015). We speculated that FGF signaling might be involved in activation of Wnt-5a-Ror2 in RCS cells, as it is known that Wnt/β-catenin and non-canonical Wnt pathways receptors can be activated by common mechanisms (Bryja et al., 2009; Grumolato et al., 2010). However, Wnt/β-catenin is likely not involved in the Noggin/Wnt-5a/Ror2 crosstalk in RCS cells because no differences in the activity analyzed by the TopFlash reporter system were observed.

Where can such FGF-dependent Noggin-induced activation of Wnt-5a-Ror2 signaling pathway in chondrocytes take place *in vivo*? In limb cartilage development, Wnt/PCP signaling appears to be involved at two steps: during condensation of cartilage elements, especially the digits (Gao et al., 2011; Wang et al., 2011; Ho et al., 2012), and for establishing cartilage growth plate morphology (Ahrens et al., 2009; Li and Dudley, 2009; Kuss et al., 2014; Romereim et al., 2014). In the first scenario, Wnt-5a is required for digit formation, and mice deficient for Wnt-5a form rudimentary digits (Yamaguchi et al., 1999). The Wnt-5a null phenotype is recapitulated by either Ror1/Ror2 double null mutants (Ho et al., 2012) or Ror2/Vangl2 double null mutants (Gao et al., 2011), clearly establishing that a Wnt-5a/Ror2/PCP pathway is necessary for digit formation. Noggin is expressed in forming cartilage condensations (Brunet et al., 1998) and could hence facilitate this process. During digit outgrowth, FGFs are expressed in the apical ectodermal ridge (AER). FGF signaling from the AER is thought to keep distal mesenchymal cells proliferating and undifferentiated (ten Berge et al., 2008). *In vitro*, FGFs inhibit chondrogenesis (Buchtova et al., 2015), but on the other hand application of FGF beads can induce ectopic digit formation *in vivo* (Montero et al., 2001). One possibility is that FGF signaling that acts at a distance from the AER on prechondrogenic cells provides competence for Noggin activity toward the Wnt-5a/Ror2/PCP pathway, and is thus enforcing PCP signaling in cells undergoing chondrogenic differentiation. In the growth plate, both Wnt-5a and Ror2 are essential for cellular polarity (Yang et al., 2003; Schwabe et al., 2004), and Wnt-5a acts via a PCP pathway (Gao et al., 2011; Kuss et al., 2014). Noggin is expressed throughout the growth plate (Brunet et al., 1998), and FGF signaling, which is a major regulator of growth plate chondrocyte proliferation, is active here as well (Horton et al., 2007).

In summary our data pinpoint a novel, yet unappreciated role for Noggin in sensitizing cells to Wnt-5a. The cellular mechanism by which Noggin accomplishes this effect on the Wnt-5a-Ror2 pathway remains to be elucidated.

## Author contributions

PK, SS, and VB designed research; MB, TR, OB, ZD, FW, AM, NC, and PK performed research; all authors analyzed data; and OB, SS, and VB wrote the paper.

### Conflict of interest statement

The authors declare that the research was conducted in the absence of any commercial or financial relationships that could be construed as a potential conflict of interest.
